# Selbstuntersuchung von Hoden und Brust – eine retrospektive Kohortenstudie an Medizinstudierenden

**DOI:** 10.1007/s00120-021-01479-8

**Published:** 2021-03-02

**Authors:** Matthias Jahnen, Lorenz Dichtl, Nora Stirenberg, Andreas Dinkel, Stefan Schiele, Helga Schulwitz, Jürgen E. Gschwend, Kathleen Herkommer

**Affiliations:** 1 Klinik und Poliklinik für Urologie, Klinikum rechts der Isar, Fakultät für Medizin, Technische Universität München, Ismaninger Str. 22, 81675 München, Deutschland; 2 Klinik und Poliklinik für Psychosomatische Medizin und Psychotherapie, Klinikum rechts der Isar, Fakultät für Medizin, Technische Universität München, Langerstr. 3, 81675 München, Deutschland

**Keywords:** Hodenkrebs, Brustkrebs, Selbstuntersuchung, Früherkennung, Sprechstunde, Testicular cancer, Self-examination, Early detection, Breast cancer, Health awareness

## Abstract

**Hintergrund:**

Durch eine regelmäßige Selbstuntersuchung kann ein Malignom der Hoden oder Brust gegebenenfalls frühzeitig erkannt und kurativ behandelt werden. Ziel dieser Arbeit war es, das Selbstuntersuchungsverhalten von Medizinstudierenden zu untersuchen und Faktoren, die eine regelmäßige Selbstuntersuchung beeinflussen, zu identifizieren.

**Methodik:**

Medizinstudierende wurden mittels Fragebogen bezüglich ihres Gesundheits- und Sexualverhaltens befragt. 98,8 % der Studierenden nahmen teil (*n* = 473). Die erhobenen Daten wurden mittels univariater und multivariater logistischer Regression analysiert.

**Ergebnisse:**

Es gaben 64,2 % der männlichen Studierenden (*n* = 177) an, regelmäßig ihre Hoden zu untersuchen und 72,2 % der weiblichen Studierenden (*n* = 296) gaben an, regelmäßig ihre Brust abzutasten. Studierende, die nicht mit ihrer/ihrem PartnerIn bzw. mit ihren FreundInnen über ihre Sexualität sprechen, führten seltener eine Selbstuntersuchung durch (*p* < 0,05). Männliche Studierende, die in den letzten 4 Wochen vor Befragung keinen Geschlechtsverkehr hatten und weibliche Studierende, die im gleichen Zeitraum nicht masturbierten, führten seltener eine Selbstuntersuchung durch (*p* < 0,05).

**Diskussion:**

Der Anteil an Medizinstudierenden, der eine regelmäßige Selbstuntersuchung durchführt, ist im Vergleich zu weniger medizinisch gebildeten jungen Erwachsenen hoch. Wissen über die Relevanz von Hoden- bzw. Brustkrebs scheint grundlegend für eine regelmäßige Selbstuntersuchung zu sein. Ein belastetes Sexualleben schränkt solch ein Gesundheitsverhalten möglicherweise ein. Eine Verbesserung der Aufklärung über Hodenkrebs und die urologische Anbindung von Jungen/junger Männer bieten daher die Möglichkeit, die Bereitschaft zur Selbstuntersuchung innerhalb dieser Altersgruppe zu steigern.

## Abstract (en)

**Background:**

Regular self-examination can facilitate early detection of testicular cancer and malignancies of the breast and may ensure a curative treatment. In this analysis we explored the tendencies of medical students to perform self-examination and associated factors.

**Methods:**

As part of their urology rotation, medical students of the Technical University of Munich were surveyed via questionnaires regarding their health and sexual behavior. In all, 98.8% of the students participated and data from 473 of 477 students were included in this analysis. Data were analyzed using univariate and multivariate regression analysis.

**Results:**

In all, 64.2% (*n* = 177) of the male students and 72.3% (*n* = 296) of the female students performed regular self-examination of the testis and breast, respectively. Students who did not communicate with their partners or friends about their sex lives were less likely to preform regular self-examination (*p* < 0.05). Male students without sexual intercourse in the 4 weeks prior to the survey and female students who did not masturbate in the 4 weeks prior to the survey were also less likely to preform regular self-examination (*p* < 0.05).

**Discussion:**

The rate of regular self-examination is high in medical students compared to previous studies on young adults and non-medical students. This shows that knowledge about the significance of testicular cancer and breast cancer are fundamental for promoting self-examination in teenagers and young adults. A distressed sex life might hinder young adults in preforming regular self-examination. Therefore, improved education about the significance of testicular cancer and routine urological consultations for male teenagers and young men are ways to promote testicular self-examination within this age group.

Malignome des Hodens sind die häufigsten bösartigen Tumorerkrankungen junger Männer im Alter von 15–39 Jahren. Jährlich erkranken in Deutschland etwa 4500 Männer an Hodenkrebs und pro Jahr sterben ca. 150 Männer als Folge der Erkrankung. Die Chance auf Heilung ist i. Allg. sehr gut und ist v. a. abhängig vom histologischen Risikoprofil und der Ausbreitung des Tumors bei Diagnose [4, 21]. Dies unterstreicht die Relevanz, Hodenkrebs in einem möglichst frühen Stadium zu diagnostizieren.

Eine neu aufgetretene Verhärtung des Hodens ist häufig das erste Symptom einer pathologischen Veränderung. Solch eine Verhärtung kann effektiv und einfach durch den betroffenen Mann selbst ertastet oder bei regelmäßigen Arztbesuchen erkannt werden, so dass die meisten Studien ein regelmäßiges Abtasten der Hoden befürworten [14]. Kampagnen wie z. B. „Hodencheck.de“ der Deutschen Gesellschaft für Urologie (DGU) zielen darauf ab, die Bereitschaft für solch eine regelmäßige Selbstuntersuchung in der Bevölkerung zu erhöhen und junge Männer zu einem regelmäßigen urologischen Arztbesuch zu animieren [13]. In unterschiedlichen Studien aus Deutschland und den USA konnte gezeigt werden, dass 36–49 % der jungen Männer im Alter von 18–35 Jahren sich selber regelmäßig abtasten oder durch einen Arzt abgetastet werden [10, 19, 25]. Es konnte außerdem gezeigt werden, dass Männer mit geringer Bildung und niedrigem sozioökonomischen Status seltener eine Selbstuntersuchung der Hoden durchführen und dass durch die öffentliche Sensibilisierung für das Thema Hodenkrebs die Bereitschaft junger Erwachsener zur Selbstuntersuchung in den vergangenen Jahrzehnten gestiegen ist [10, 19]. Dennoch sind Jungen und junge Männer hinsichtlich Hodenkrebs häufig unzureichend informiert und in den meisten Fällen auch nicht an einen Urologen angebunden, so dass eine individuelle Beratung bzgl. Vorsorge, Früherkennung und urologischer Gesundheit oftmals unterbleibt [19].

Im Vergleich dazu ist aufgrund von Vorsorgeuntersuchungen im Rahmen der Zervixkarzinomprävention und der Verschreibungspflicht hormoneller Kontrazeptiva die frauenärztliche Anbindung junger Frauen häufiger gegeben [5]. Diese regelmäßigen Arztbesuche ermöglichen es jungen Frauen außerdem, frühzeitig hinsichtlich Brustkrebs und regelmäßiger Selbstuntersuchung der Brust beraten zu werden. Zahlen zum Selbstuntersuchungsverhalten junger Frauen in Deutschland ebenso wie eine allgemeingültige Empfehlung zur Selbstuntersuchung der Brust als Früherkennungsmaßnahme fehlen aber [6, 15].

Es ist wichtig, junge Erwachsene zu motivieren, sich eigenständig zu untersuchen und für das Thema Hoden- bzw. Brustkrebs zu sensibilisieren, um die Rate an frühdiagnostizierten, kurativ behandelbaren Tumoren zu steigern. Diesbezüglich scheint die öffentliche Informationsverbreitung mittels Aufklärungskampagnen ein wichtiger Schritt. Inwiefern darüber hinaus aber andere Faktoren, insbesondere sexuelle Aufgeschlossenheit, Sexualkundeunterricht und das Sexualleben, die Bereitschaft für eine regelmäßige Selbstuntersuchung beeinflussen können, ist zum Großenteil noch unklar. In dieser Analyse wurde das Selbstuntersuchungsverhalten einer medizinisch gebildeten Kohorte ermittelt und Faktoren, welche im Zusammenhang mit regelmäßiger Selbstuntersuchung der Hoden oder Brust stehen, identifiziert.

## Studiendesign und Untersuchungsmethoden

### Design und Prozedere

Bei der vorliegenden Studie handelt es sich um eine Querschnittsuntersuchung mit einer anfallenden Stichprobe. Im Rahmen der medizinischen Lehre der Klinik und Poliklinik für Urologie des Klinikums rechts der Isar wurden Medizinstudierende der Technischen Universität München des 4. und 5. Studienjahrs während des Praktikumstags (April 2018 bis Februar 2020) mittels anonymisierter Fragebögen bezüglich ihres Gesundheits- und Sexualverhaltens befragt. Die Fragebögen für männliche und weibliche Studierende waren bis auf einzelne geschlechtsspezifische Fragen gleich. In diese Analyse wurden alle Studierenden eingeschlossen, die die Frage zur Selbstuntersuchung beantwortet hatten. Die Ethikkommissen der Technischen Universität München hat der vorliegenden Studie zugestimmt.

### Instrumente

Zur Erfassung des Selbstuntersuchungsverhaltens wurden die männlichen Studierenden gefragt, ob sie regelmäßig ihre Hoden untersuchen bzw. die weiblichen Studierenden gefragt, ob sie regelmäßig ihre Brust untersuchen (nein; ja).

Die folgenden soziodemografischen Faktoren und Lebensstilfaktoren wurden in die Analyse eingeschlossen: Alter (*Jahre*), Wohnsituation (bei den Eltern; Wohngemeinschaft; mit PartnerIn/Familie; Wohnheim; eigene Wohnung), Partnerschaft (nein; ja; ja, Fernbeziehung), Kinder (nein; ja), BMI (kg/m^2^), Zufriedenheit mit dem eigenen Aussehen (Skala von 1 [sehr unzufrieden] bis 10 [sehr zufrieden]).

Des Weiteren wurden die Studierenden gefragt, ob sie mit ihrer/ihrem PartnerIn, ihren Eltern, ihren FreundInnen oder mit Fremden über die eigene sexuelle Aktivität kommunizieren (nein; ja). Die Teilnahme an einem Sexualkundeunterricht zur Schulzeit wurde erhoben (nein; ja). Außerdem wurde erfasst, ob die Studierenden die Notwendigkeit für mehr Aufklärungsunterricht in der Schule sehen (nein; ja).

Hinsichtlich sexueller Orientierung und des Sexualverhaltens wurden folgende Parameter in die Analyse eingeschlossen: sexuelle Orientierungsidentität (heterosexuell; eher heterosexuell; bisexuell; eher homosexuell; homosexuell), sexuell bevorzugtes Geschlecht (weiblich; männlich; anderes), vaginaler Geschlechtsverkehr (jemals im Leben; [nein; ja]), vaginaler Geschlechtsverkehr (in den letzten 4 Wochen; nein; ja), Masturbationshäufigkeit in den letzten 4 Wochen, Verwendung von Kondomen (im letzten Jahr; nein; ja), Gebrauch einer Notfallkontrazeption (im letzten Jahr; nein; ja), Nachweis einer sexuell übertragbaren Erkrankung (jemals im Leben; nein; ja). Die männlichen Studierenden wurden außerdem gefragt, ob sie unter einer eigeschränkten Erektionsfähigkeit (Penis bei der Erektion nicht komplett hart), an einem frühzeitigen Samenerguss (innerhalb einer 1 min nach vaginaler Penetration) oder an Schmerzen während des Geschlechtsverkehrs leiden (jeweils: nein; ja). Das Beantworten einer oder mehrerer dieser Fragen mit „ja“ wurde als mögliche Beeinträchtigung der sexuellen Funktion definiert.

### Statistische Auswertung

Zur Beschreibung der Stichprobe erfolgte die Darstellung kategorialer Parameter in absoluten Zahlen und Prozent. Stetige Parameter wurden als Mittelwert plus Standardabweichung bzw. Median plus Interquartilsbereich dargestellt. Assoziationen zwischen den erhobenen Parametern und dem Selbstuntersuchungsverhalten männlicher sowie weiblicher Studierender wurde mittels logistischer Regressionsanalyse berechnet. *P*-Werte ≤ 0,05 wurden als signifikant definiert. Die statistische Auswertung erfolgte mittels SAS (Version 9.4, SAS Institute Inc, Cary, NC, USA).

## Ergebnisse

Die Fragebögen wurden von 98,9 % aller (*n* = 482) Medizinstudierenden, die am Praktikumstag teilnahmen, ausgefüllt; 4 Studierende wurden aufgrund eines unzureichend ausgefüllten Fragebogens ausgeschlossen. Insgesamt wurden 473 durchschnittlich 25 Jahre alte (MW = 24,9 Jahre, SD = 2,9 Jahre) Medizinstudierende in diese Analyse eingeschlossen (177 männliche und 296 weibliche Studierende; Tab. 1).

**Table 1:** 

	Männliche Studierende (*n* = 177)		Weibliche Studierende (*n* = 296)		Gesamtkohorte (*n* = 473)	
	*n*	%	*n*	%	*n*	%
**Regelmäßige Selbstuntersuchung** (Hoden/Brust)						
Nein	63	35,8	82	27,8	145	30,8
Ja	113	64,2	213	72,2	326	69,2
**Soziodemographie und Lebensstilfaktoren**						
Alter (Jahre) MW (SD)	25,1 (3,2)		24,8 (2,8)		24,9 (2,9)	
BMI (kg/m^2^) MW (SD)	23,4 (2,7)		21,0 (2,8)		21,9 (3,0)	
Zufriedenheit mit dem eigenen Aussehen (1–10) MW (SD)	7,3 (1,5)		7,0 (1,7)		7,1 (1,6)	
*Wohnsituation*						
Bei den Eltern	9	5,1	26	8,9	35	7,5
Wohngemeinschaft	74	42,3	121	41,3	195	41,7
Mit PartnerIn/Familie	31	17,7	55	18,8	86	18,4
Wohnheim	14	8,0	20	6,8	34	7,3
Eigene Wohnung	47	26,9	71	24,2	118	25,2
*Partnerschaft*						
Nein	63	36,0	92	31,2	155	33,0
Ja	99	56,6	185	62,7	284	60,4
Fernbeziehung	13	7,4	18	6,1	31	6,6
*Kinder*						
Nein	165	95,9	287	98,3	452	97,4
Ja	7	4,1	5	1,7	12	2,6
**Sexuelle Kommunikation und Bildung**						
*Kommunikation über Sexualität mit PartnerIn*						
Nein/Nein (kein/e PartnerIn)	7	4,0	17	5,8	24	5,1
Ja/Ja (kein/e PartnerIn)	168	96,0	277	94,2	445	94,9
*Kommunikation über Sexualität mit den Eltern*						
Nein	144	82,3	234	79,6	378	80,6
Ja	31	17,7	60	20,4	91	19,4
*Kommunikation über Sexualität mit FreundInnen*						
Nein	42	23,9	42	14,3	84	17,9
Ja	134	76,1	252	85,7	386	82,1
*Kommunikation über Sexualität mit Fremden*						
Nein	147	84,0	263	89,5	410	87,4
Ja	28	16,0	31	10,5	59	12,6
*Sexualkundeunterricht in der Schule oder dem Studium*						
Nein	48	27,3	78	26,9	126	27,0
Ja	128	72,7	212	73,1	340	73,0
*Notwendigkeit für mehr Aufklärung in der Schule*						
Nein	47	27,0	41	14,1	88	19,0
Ja	127	73,0	249	85,9	376	81,0
**Sexualleben und sexuelle Gesundheit**						
*Sexuelle Orientierungsidentität*						
Heterosexuell	145	82,9	227	77,5	372	79,5
Eher heterosexuell	18	10,3	55	18,8	73	15,6
Bisexuell	3	1,7	8	2,7	11	2,4
Eher homosexuell/homosexuell	9	5,1	3	1,0	12	2,6
*Sexuell bevorzugtes Geschlecht*						
Weiblich	158	90,8	12	4,2	170	36,8
Männlich	15	8,6	276	95,8	291	63,0
Anderes	1	0,6	0	0,0	1	0,2
*Vaginaler Geschlechtsverkehr (jemals im Leben)*						
Nein	15	8,6	25	8,6	40	8,6
Ja	160	91,4	267	91,4	427	91,4
*Vaginaler Geschlechtsverkehr (in den letzten 4 Wochen)*						
Nein	49	28,3	74	25,7	123	26,7
Ja	124	71,7	214	74,3	338	73,3
Masturbationshäufigkeit (in den letzten 4 Wochen; [*n*] M [IQR])	10 (6–20)		3 (1–5)		5 (2–8)	
*Verwendung von Kondomen (im letzten Jahr)*						
Nein	54	30,7	116	39,3	170	36,1
Ja	122	69,3	179	60,7	301	63,9
*Notfallkontrazeption *(im letzten Jahr)						
Nein	162	92,1	263	89,2	425	90,2
Ja	14	7,9	32	10,8	46	9,8
*Sexuell übertragbare Erkrankung (jemals im Leben)*						
Nein	145	82,9	250	85,0	395	84,2
Ja	30	17,1	44	15,0	74	15,8
*Beeinträchtigte sexuelle Funktion*						
Nein	149	84,7	–		–	
Ja	27	15,3	–		–	

*MW* Mittelwert, *SD* Standardabweichung, *M* Median, *IQR* Interquartilsbereich, *BMI* Body-Mass-Index

**Figure 1:**
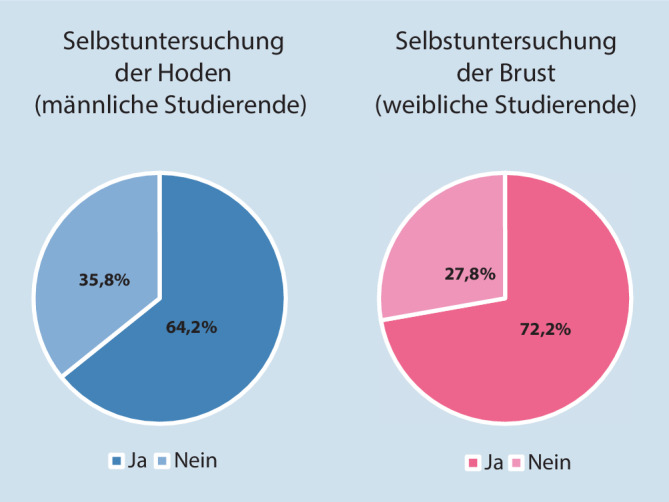

Es gaben 64,2 % der männlichen Studierenden an, regelmäßig ihre Hoden zu untersuchen und 72,2 % der weiblichen Studierenden gaben an, regelmäßig ihre Brust abzutasten (Abb. 1).

Mehr als 90,0 % der männlichen sowie weiblichen Studierenden gaben an, mit ihrem/ihrer PartnerIn über die eigene Sexualität zu sprechen bzw. zu sprechen, wenn sie eine/einen hätten. Kommunikation über Sexualität mit FreundInnen wurde von mehr als drei Viertel der Studierenden angegeben (männliche Studierende: 76,1 % vs. weibliche Studierende: 85,7 %). Jeder fünfte der Studierenden gab an, mit seinen/ihren Eltern über die eigene Sexualität zu sprechen und nur eine Minderheit der Studierenden gab an, mit Fremden über die eigene Sexualität zu sprechen (12,6 %). Zwar gaben mehr als zwei Drittel der männlichen und weiblichen Studierenden an, an Sexualkundeunterricht in der Schule teilgenommen zu haben, aber die meisten Studierenden sahen trotzdem die Notwendigkeit für mehr Aufklärung in der Schule (Tab. 1).

Die meisten Studierenden gaben an, eine heterosexuelle bzw. eine eher heterosexuelle Orientierungsidentität zu haben. Die meisten Studierenden waren sexuell erfahren und gaben an, schon einmal vaginalen Geschlechtsverkehr im Leben (91,4 %) bzw. in den letzten 4 Wochen gehabt zu haben (73,3 %). Bei 15,3 % der männlichen Studierenden zeigte sich der Hinweis auf eine beeinträchtigte sexuelle Funktion (Tab. 1).

**Figure 2:**
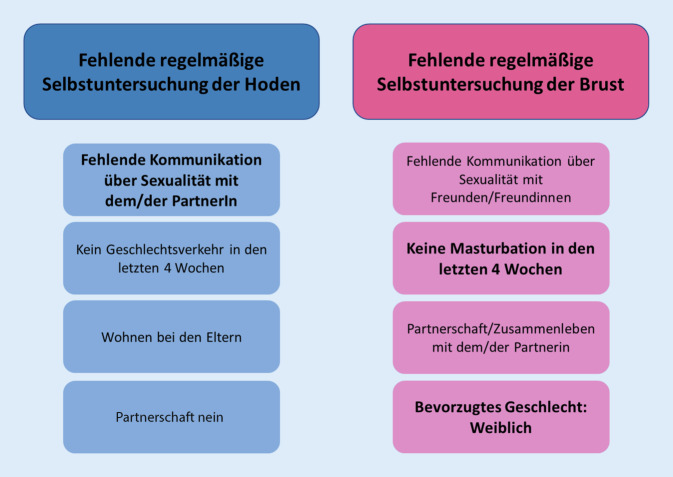

In der univariaten Analyse zeigten sich einzelne, für weibliche und männliche Studierende spezifische Assoziationen mit einer regelmäßigen Selbstuntersuchung der Hoden bzw. Brust. Männliche Studierende, die angaben, nicht mit ihrer/ihrem PartnerIn über die eigene Sexualität zu sprechen, führten seltener eine Selbstuntersuchung durch (*p* < 0,05). Eine ähnliche Assoziation zeigte sich bei weiblichen Studierenden, die eine fehlende sexuelle Kommunikation mit FreundInnen angaben (*p* < 0,05; Tab. 2 und Abb. 2).

**Table 2:** 

	Männliche Studierende		Weibliche Studierende	
Charakteristika	OR (95 %-KI)	p	OR (95 %-KI)	*p*
**Soziodemographie und Lebensstilfaktoren**				
Alter (stetig)	0,96 (0,87–1,06)	0,44	1,00 (0,91–1,10)	0,99
BMI (stetig)	0,98 (0,88–1,10)	0,73	1,00 (0,91–1,09)	0,94
Zufriedenheit mit dem eigenen Aussehen (stetig)	1,06 (0,86–1,30)	0,58	0,98 (0,85–1,14)	0,79
*Partnerschaft (Ref. nein)*				
Ja	2,06 (1,07–3,98)	0,03	0,47 (0,26–0,86)	**0,01**
Fernbeziehung	1,37 (0,40–4,63)	0,93	4,14 (0,52–33,1)	0,08
*Kinder (Ref. ja)*				
Nein	0,70 (0,13–3,72)	0,68	0,65 (0,07–5,88)	0,70
*Wohnsituation (Ref. eigene Wohnung)*				
Bei den Eltern	0,13 (0,03–0,72)	**0,02**	1,35 (0,40–4,55)	0,63
Wohngemeinschaft	0,92 (0,42–2,00)	0,83	0,60 (0,30–1,22)	0,16
Mit PartnerIn/Familie	1,35 (0,49–3,71)	0,56	0,43 (0,19–0,96)	**0,04**
Wohnheim	0,35 (0,10–1,20)	0,09	0,37 (0,13–1,07)	0,07
**Sexuelle Kommunikation und Bildung**				
*Kommunikation über Sexualität mit PartnerIn (Ref. ja/ja; kein/e PartnerIn)*				
Nein/Nein (kein/e PartnerIn)	0,08 (0,01–0,73)	**0,02**	0,40 (0,15–1,08)	0,07
*Kommunikation über Sexualität mit den Eltern (Ref. ja)*				
Nein	0,56 (0,24–1,35)	0,20	0,68 (0,34–1,33)	0,26
*Kommunikation über Sexualität mit FreundInnen (Ref. ja)*				
Nein	0,67 (0,33–1,37)	0,28	0,44 (0,23–0,87)	**0,02**
*Kommunikation über Sexualität mit Fremden (Ref. ja)*				
Nein	0,67 (0,27–1,62)	0,37	1,07 (0,47–2,42)	0,88
Sexualkundeunterricht in der Schule oder dem Studium* (Ref. ja)*				
Nein	1,16 (0,58–2,37)	0,67	0,79 (0,45–1,40)	0,41
Notwendigkeit für mehr Aufklärung in der Schule *(Ref. ja)*				
Nein	0,54 (0,27–1,07)	0,08	1,97 (0,84–4,66)	0,12
**Sexualleben und sexuelle Gesundheit**				
*Sexuelle Orientierungsidentität (Ref. heterosexuell)*				
Eher heterosexuell	0,40 (0,15–1,07)	0,07	1,61 (0,78–3,30)	0,20
Bisexuell	0,99 (0,09–11,2)	0,99	0,67 (0,16–2,88)	0,59
Eher homosexuell/homosexuell	0,62 (0,16–2,41)	0,49	0,80 (0,07–9,00)	0,86
*Sexuell bevorzugtes Geschlecht (Ref. weiblich)*				
Männlich	0,59 (0,20–1,72)	0,34	–	–
*Sexuell bevorzugtes Geschlecht (Ref. männlich)*				
Weiblich	–	–	0,35 (0,11–1,13)	0,08
*Vaginaler Geschlechtsverkehr (jemals im Leben; Ref. ja)*				
Nein	0,46 (0,16–1,33)	0,15	0,80 (0,33–1,93)	0,62
*Vaginaler Geschlechtsverkehr (in den letzten 4 Wochen; Ref. ja)*				
Nein	0,48 (0,24–0,94)	**0,03**	1,27 (0,69–2,33)	0,44
*Masturbationshäufigkeit (in den letzten 4 Wochen) (stetig)*	0,99 (0,97–1,03)	0,92	–	–
*Masturbation (in den letzten 4 Wochen; Ref. ja)*				
Nein	–	–	0,45 (0,26–0,79)	**0,006**
*Verwendung von Kondomen *(im letzten Jahr) *(Ref. ja)*				
Nein	1,17 (0,60–2,29)	0,65	1,02 (0,60–1,72)	0,94
*Notfallkontrazeption (im letzten Jahr; Ref. nein)*				
Ja	1,43 (0,43–4,77)	0,56	0,71 (0,32–1,54)	0,38
*Sexuell übertragbare Erkrankung (jemals im Leben; Ref. nein)*				
Ja	0,97 (0,43–2,19)	0,93	0,62 (0,31–1,21)	0,16
*Beeinträchtigte sexuelle Funktion (Ref. nein)*				
Ja	0,46 (0,20–1,04)	0,06	–	–

*Ref.* Referenz, *KI* Konfidenzintervall, *OR* Odds Ratio, *BMI* Body-Mass-Index

Außerdem führten männliche Studierende, die bei ihren Eltern wohnten und jene, die keinen vaginalen Geschlechtsverkehr in den letzten 4 Wochen hatten, signifikant seltener eine regelmäßige Selbstuntersuchung der Hoden durch (*p* < 0,05). Auch zeigte sich der Trend, dass männliche Studierende mit dem Hinweis auf eine eingeschränkte sexuelle Funktion seltener eine regelmäßige Selbstuntersuchung der Hoden durchführten (*p* = 0,06; Tab. 2 und Abb. 2).

Bei weiblichen Studierenden zeigte sich eine signifikante Assoziation zwischen einer Partnerschaft bzw. dem Zusammenleben mit PartnerIn/Familie und dem Unterlassen einer regelmäßigen Selbstuntersuchung der Brust (*p* < 0,05; Tab. 2 und Abb. 2).

Es zeigten sich bei Studierenden beider Geschlechter keine signifikanten Assoziationen zwischen der Teilnahme an einem Sexualkundeunterricht und einer regelmäßigen Selbstuntersuchung der Hoden bzw. Brust (Tab. 2).

In der multivariaten Regression zeigte sich bei männlichen Studierenden eine signifikante Assoziation zwischen fehlender Kommunikation mit dem/der PartnerIn und fehlender Selbstuntersuchung der Hoden (OR: 0,11 [0,02–0,95]). Bei weiblichen Studieren zeigte sich in der multivariaten Analyse, dass insbesondere weibliche Studierende, die in den letzten 4 Wochen nicht masturbiert hatten (OR: 0,43 [0,24–0,78]) und jene, die das weibliche als sexuell bevorzugtes Geschlecht angaben (OR: 0,26 [0,07–0,96]), seltener eine Selbstuntersuchung der Brust durchführen (alles *p* < 0,05; Abb. 2).

## Diskussion

Eine regelmäßige Selbstuntersuchung der Hoden ermöglicht es jungen Männern, eine Veränderung der Hoden frühzeitig zu erkennen, sodass ein mögliches Malignom in einem frühen Stadium diagnostiziert und behandelt werden kann [11, 14]. Der regelmäßigen Durchführung solch einer Selbstuntersuchung scheinen insbesondere Wissen über Hodenkrebs und ein hohes Bildungsniveau zugrunde zu liegen [10, 19, 24]. In unserer Studie gaben 64,2 % der befragten männlichen Medizinstudierenden an, regelmäßig ihre Hoden zu untersuchen. Dieser Anteil liegt damit deutlich höher als in vorangegangenen Studien. In Studien aus den USA und Irland gaben 36,0 % der Männer im Alter von 18–35 Jahren bzw. 18–67 Jahren an mehrmals im Jahr eine Selbstuntersuchung der Hoden durchzuführen [8, 10, 25]. In einer Studie an deutschen Studierenden gaben 48,9 % der männlichen Teilnehmer an, solch eine Untersuchung schon einmal durchgeführt zu haben. Im Vergleich mit diesen Studien ist es wichtig zu beachten, dass in unsere Analyse ausschließlich Daten von Medizinstudierenden des 4. bis 5. Studienjahrs, die bereits an der Urologie-Vorlesung teilgenommen hatten, eingeflossen sind. In diesem Kollektiv kann man daher davon ausgehen, dass das Wissen über Hodenkrebs, Vorsorge und das Verständnis für Gesundheitsverhalten höher ist als in der Allgemeinbevölkerung und auch im Vergleich zu Studierenden anderer Fächer [8, 23]. Dementsprechend unterstreichen die Ergebnisse dieser Analyse, dass ein hohes Bildungsniveau an sich und insbesondere spezifisches medizinisches Wissen die Bereitschaft zur regelmäßigen Selbstuntersuchung bestärken kann.

Ausreichendes Wissen über Hodenkrebs und dessen Relevanz ist bei vielen Jugendlichen und jungen Erwachsenen ohne medizinischen Hintergrund aber nicht gegeben, sodass diesen die Grundlage für das Verständnis für die Durchführung einer regelmäßige Selbstuntersuchung der Hoden fehlt [1, 16, 17]. Die Entwicklungen der letzten Jahrzehnte deuten aber darauf hin, dass durch öffentliche Wissensvermittlung die Kenntnis der Allgemeinbevölkerung über Hodenkrebs angestiegen ist und auch das Selbstuntersuchungsverhalten gesteigert werden kann [10, 20]. Zusammengenommen zeigt dies, dass es sinnvoll ist, weitere Bemühungen mit der Absicht, Jungen und junge Männer bezüglich Hodenkrebs zu informieren und zur Durchführung einer regelmäßigen Selbstuntersuchung der Hoden zu motivieren. Zum Aufbau eines angemessenen Wissensstands über Hodenkrebs können unterschiedliche Institutionen als mögliche Informationsquelle dienen. Vorangegangene Arbeiten konnten bereits zeigen, dass durch die Informationsverbreitung mit Hilfe von Aufklärungskampagnen das Wissen über Hodenkrebs und die Bereitschaft zur Selbstuntersuchung der Hoden gesteigert werden kann, aber die Reichweite dieser Kampagnen häufig beschränkt ist [10, 19]. Die adäquate Anbindung solcher Kampagnen an soziale Netzwerke wäre ein nächster wichtiger Schritt, um die Zielgruppe der Jungen und jungen Männer besser zu erreichen.

Als eine weitere Informationsquelle für Jungen und junge Männer können ÄrztInnen dienen. Die zweite Jugenduntersuchung (J2) durch den/die Kinderarzt/Kinderärztin im Alter von 16–18 Jahren bietet die Möglichkeit, Gesundheitsvorsorge i. Allg. anzusprechen. Diese Jugenduntersuchung wird aber nur von einem Fünftel der Jugendlichen wahrgenommen und legt den Fokus meist nur unzureichend auf urologische Themen [22]. Der direkte Kontakt zu einem/einer Urologen/Urologin wird von den meisten jungen Männern nur bei akuten Problemen gesucht. Im Gegensatz dazu ist die Anbindung von jungen Frauen an einen/eine Gynäkologen/Gynäkologin deutlich häufiger gegeben, so dass Fragen zu Themen wie Verhütung, sexuell übertragbare Erkrankungen und Krankheiten des Unterleibs sowie der Brust beantwortet werden können [9, 18]. Dies trägt möglicherweise dazu bei, dass im Vergleich zu gleichaltrigen Männern Frauen der eigenen Gesundheit häufig mehr Aufmerksamkeit schenken und auch das Bewusstsein bzgl. der Relevanz von Brutkrebs in der Gesellschaft weiter verbreitet ist als Wissen über Hodenkrebs [12]. Zusammengenommen könnte dies dazu führen, dass bereits junge Frauen, obwohl sie nur ein sehr geringes Risiko haben an Brustkrebs zu erkranken, eine hohe Bereitschaft haben, die eigene Brust zu untersuchen. Dies spiegelt sich auch in den Ergebnissen dieser Analyse wider, die zeigen, dass mehr weibliche Studierende (72,2 %) eine regelmäßige Selbstuntersuchung der Brust durchführen als männliche Studierende (64,2 %) eine Selbstuntersuchung der Hoden. Um eine ärztliche Versorgung junger Männer ähnlich der von jungen Frauen zu gewährleisten, ist es Ziel der DGU und des Berufsverbandes der Deutschen Urologen (BvDU), eine „Jungen-Sprechstunde“ für Jungen und junge Männer zu etablieren. In solch einem Rahmen könnte ergänzend zur unpopulären J2-Untersuchung eine Beratung hinsichtlich urologischer Gesundheit und eine Anleitung zur Selbstuntersuchung erfolgen und so das Gesundheitsverhalten junger Männer gefördert werden [3]. In Deutschland gibt es bisher eine private Krankenkasse, die einen Sondervertrag zur Hodenkrebsfrüherkennung mit der BvDU abgeschlossen hat [2]. Solche Verträge sollten in Zukunft weiter unterstützt werden, um eine urologische Anbindung junger Männer zu verbessern.

Trotz vergleichbaren Wissensstands gab jeder dritte männliche Studierende an, keine regelmäßige Selbstuntersuchung der Hoden durchzuführen und jede vierte weibliche Studierende gab an, keine regelmäßige Selbstuntersuchung der Brust durchzuführen. Dies deutet darauf hin, dass es weitere Faktoren neben dem Kenntnisstand über Hoden- bzw. Brustkrebs gibt, die das Selbstuntersuchungsverhalten beeinflussen können. Solche Faktoren bieten möglicherweise weitere Ansatzpunkte, junge Erwachsene zu einem bewussteren Gesundheitsverhalten und dem Durchführen einer regelmäßigen Selbstuntersuchung zu motivieren. In vorangegangenen Studien wurde bereits in einem Kollektiv von Männern im Alter von 18–35 Jahren festgestellt, dass mit steigendem Alter die Bereitschaft zur Selbstuntersuchung der Hoden zunimmt [20, 25]. Solch eine Assoziation mit dem Alter konnten wir aufgrund der schmalen Altersverteilung (MW: 24,9 ± 2,9 Jahre) in unserer Studie nicht darstellen. Es konnte jedoch gezeigt werden, dass männliche Studierende, die noch bei ihren Eltern wohnen, weniger häufig eine Selbstuntersuchung durchführen. Diese Ergebnisse deuten darauf hin, dass eine regelmäßige Selbstuntersuchung nicht unbedingt ein gewisses Alter, sondern evtl. auch ein gewisses Maß an Selbstständigkeit erfordert. Daher ist es wichtig, bereits Jungen die Verantwortung über ihre eigene Gesundheit bewusst zu machen, um Gesundheitsverhalten wie eine regelmäßige Selbstuntersuchung der Hoden zu fördern.

Des Weiteren sind in dieser Analyse insbesondere Faktoren, die das Sexualverhalten und die sexuelle Aufgeschlossenheit der Studierenden widerspiegeln, berücksichtigt worden. So konnte gezeigt werden, dass männliche Studierende, die nicht mit ihrem/ihrer PartnerIn über die eigene Sexualität sprechen, seltener eine Selbstuntersuchung der Hoden durchführen. Auch weibliche Studierende, die nicht mit ihren FreundInnen über ihre Sexualität sprechen, gaben an, seltener eine Selbstuntersuchung der Brust durchzuführen. Das Verzichten auf solch eine Kommunikation mit dem/der PartnerIn oder FreundInnen kann auf eine gewisse Unsicherheit in Bezug auf die eigene Sexualität hinweisen. Dies geht einher mit einer britischen Studie, die gezeigt hat, dass junge Frauen mit höherer selbstwahrgenommener sexueller Attraktivität häufiger eine Selbstuntersuchung durchführen [7]. Solch eine Zurückhaltung im Umgang mit der eigenen Sexualität scheint sowohl jungen Männern als auch junge Frauen in der Bereitschaft zur Selbstuntersuchung, die den aktiven Umgang mit dem eigenen Körper und insbesondere intimer Zonen erfordert, einzuschränken. Dass das Sexualleben möglicherweise Einfluss auf das Selbstuntersuchungsverhalten junger Erwachsener haben kann, zeigt sich auch daran, dass männliche Studierende, die angaben, in den letzten 4 Wochen keinen Geschlechtsverkehr gehabt zu haben und weibliche Studierende, die angaben, in den letzten 4 Wochen nicht masturbiert zu haben, seltener eine Selbstuntersuchung durchführten. Außerdem war in der univariaten Analyse der Trend zu erkennen, dass männliche Studierende, bei denen sich Hinweise auf eine eingeschränkte sexuelle Funktion zeigten, weniger häufig eine regelmäßige Selbstuntersuchung durchführten. Dies deutet darauf hin, dass ein möglicherweise unerfülltes ggf. sogar belastetes Sexualleben einen negativen Einfluss auf das Selbstuntersuchungsverhalten junger Erwachsener haben kann. In diesem Kontext erscheint es umso wichtiger, junge Männer dazu zu motivieren bei sexuellen Problemen den Kontakt zum/zur Urologen/Urologin zu suchen, um die sexuelle Gesundheit zu fördern und so auch positiv auf das allgemeine urologische Gesundheitsverhalten einzuwirken. Durch die Etablierung der bereits oben erwähnten urologischen „Jungen-Sprechstunde“ könnte solch eine Selbstverständlichkeit, sich bei sexuellen Problemen an einen/eine Urologen/Urologin zu wenden, gefördert werden und das Gesundheitsbewusstsein auf unterschiedlichen Ebenen nachhaltig positiv beeinflusst werden.

Zusammenfassend deuten die Ergebnisse dieser Analyse daraufhin, dass es notwendig ist, Jugendliche und junge Erwachsene umfangreich bezüglich sexueller Gesundheit und Hodenkrebs bzw. Brustkrebs aufzuklären sowie sie zu gesundheitsbezogener Selbstständigkeit zu ermutigen, um Gesundheitsverhalten wie das regelmäßige Abtasten von Hoden und Brust zu fördern. Dies sollte bereits in einem gewissen Ausmaß in der Schule und später durch unterschiedliche Bezugspersonen vermittelt werden. Besonders der Kontakt junger Männer mit einem Urologen, über die Versorgung akuter Probleme hinaus sollte z. B. durch eine „Jungen-Sprechstunde“ gefördert werden, um Themen wie Hodenkrebs, Früherkennung und urologische Gesundheitsvorsorge ins Bewusstsein dieser Altersgruppe zu rufen.

Bei Betrachtung der Ergebnisse dieser Arbeit müssen unterschiedliche Gesichtspunkte in Betracht gezogen werden. Zum einen handelt sich bei dem Kollektiv um Medizinstudierende, sodass sich die Ergebnisse nicht zwangsläufig auf junge Erwachsene mit anderem Bildungshintergrund übertragen lassen. Des Weiteren wurde die sexuelle Funktion männlicher Studierender nur mit einzelnen Fragen erhoben und ist nicht mit einer klinischen Diagnose gleich zusetzten. Dies ist außerdem nach unserem Kenntnisstand die erste Studie, die den Zusammenhang zwischen dem Sexualverhalten und dem Selbstuntersuchungsverhalten junger Erwachsener untersucht hat, so dass Vergleichsdaten fehlen. Aufgrund der hohen Beteiligungsrate sind die erhobenen Daten aber als sehr valide in Bezug auf deutsche Medizinstudierende einzuschätzen.

## Fazit für die Praxis

- Ein Großteil von Medizinstudierenden führen eine regelmäßige der Selbstuntersuchung der Hoden bzw. Brust durch.
- Ein belastetes Sexualleben kann die Bereitschaft junger Männer zur Selbstuntersuchung der Hoden einschränken.
- Wissen über die Relevanz von Hodenkrebs sollte in der Schule, durch adäquate Hodenkrebskampagnen und Ärzte vermittelt werden.
- Die urologische Anbindung von Jungen und jungen Männern sollte gefördert werden, um diese hinsichtlich der Früherkennung von Hodentumoren und möglicher sexueller Probleme zu beraten.
