# Erratum zu: Selbstuntersuchung von Hoden und Brust – eine retrospektive Kohortenstudie an Medizinstudierenden

**DOI:** 10.1007/s00120-021-01580-y

**Published:** 2021-06-29

**Authors:** Matthias Jahnen, Lorenz Dichtl, Nora Stirenberg, Andreas Dinkel, Stefan Schiele, Helga Schulwitz, Jürgen E. Gschwend, Kathleen Herkommer

**Affiliations:** 1 Klinik und Poliklinik für Urologie, Klinikum rechts der Isar, Fakultät für Medizin, Technische Universität München, Ismaninger Str. 22, 81675 München, Deutschland; 2 Klinik und Poliklinik für Psychosomatische Medizin und Psychotherapie, Klinikum rechts der Isar, Fakultät für Medizin, Technische Universität München, Ismaninger Str. 22, 81675 München, Deutschland

**Erratum zu:**

**Urologe 2021**

10.1007/s00120-021-01479-8

In der zunächst online publizierten Version dieses Artikels sind Fehler in den Tabellen 1 und 2 und der Abb. 1 aufgetreten. Der Beitrag wurde korrigiert und wir bitten Sie, die korrekte Version des Artikels zu beachten.

